# Supplementary material for: HIVprotI: an integrated web based platform for prediction and design of HIV proteins inhibitors
Source: J Cheminform. 2018 Mar 9;10:12. doi: 10.1186/s13321-018-0266-y (PMC5845081; doi:10.1186/s13321-018-0266-y)
Supplement: Supplementary file 2 — Additional file 2. Source code of HIVProtI web server. [file 13321_2018_266_MOESM2_ESM.zip › HIVprotI_Source-code/hivprotI/weka-3-6-6/doc/weka/filters/unsupervised/instance/package-summary.html]

weka.filters.unsupervised.instance


---


|  |  |  |
| --- | --- | --- |
| **Overview** \| **Package** \| Class \| **Tree** \| **Deprecated** \| **Index** \| **Help** \| **Weka's home** | |  |
| **PREV PACKAGE**   **NEXT PACKAGE** | **FRAMES**    **NO FRAMES**     **All Classes** |  |


---

## Package weka.filters.unsupervised.instance

| **Class Summary** | |
| --- | --- |
| **NonSparseToSparse** | An instance filter that converts all incoming instances into sparse format. |
| **Normalize** | An instance filter that normalize instances considering only numeric attributes and ignoring class index. |
| **Randomize** | Randomly shuffles the order of instances passed through it. |
| **RemoveFolds** | This filter takes a dataset and outputs a specified fold for cross validation. |
| **RemoveFrequentValues** | Determines which values (frequent or infrequent ones) of an (nominal) attribute are retained and filters the instances accordingly. |
| **RemoveMisclassified** | A filter that removes instances which are incorrectly classified. |
| **RemovePercentage** | A filter that removes a given percentage of a dataset. |
| **RemoveRange** | A filter that removes a given range of instances of a dataset. |
| **RemoveWithValues** | Filters instances according to the value of an attribute. |
| **Resample** | Produces a random subsample of a dataset using either sampling with replacement or without replacement. |
| **ReservoirSample** | Produces a random subsample of a dataset using the reservoir sampling Algorithm "R" by Vitter. |
| **SparseToNonSparse** | An instance filter that converts all incoming sparse instances into non-sparse format. |
| **SubsetByExpression** | Filters instances according to a user-specified expression.    Grammar:    boolexpr\_list ::= boolexpr\_list boolexpr\_part \| boolexpr\_part;    boolexpr\_part ::= boolexpr:e {: parser.setResult(e); :} ;    boolexpr ::= BOOLEAN   \| true  \| false  \| expr < expr  \| expr <= expr  \| expr > expr  \| expr >= expr  \| expr = expr  \| ( boolexpr )  \| not boolexpr  \| boolexpr and boolexpr  \| boolexpr or boolexpr  \| ATTRIBUTE is STRING  ;    expr ::= NUMBER  \| ATTRIBUTE  \| ( expr )  \| opexpr  \| funcexpr  ;    opexpr ::= expr + expr  \| expr - expr  \| expr \* expr  \| expr / expr  ;    funcexpr ::= abs ( expr )  \| sqrt ( expr )  \| log ( expr )  \| exp ( expr )  \| sin ( expr )  \| cos ( expr )  \| tan ( expr )  \| rint ( expr )  \| floor ( expr )  \| pow ( expr for base , expr for exponent )  \| ceil ( expr )  ;    Notes:  - NUMBER  any integer or floating point number   (but not in scientific notation!)  - STRING  any string surrounded by single quotes;   the string may not contain a single quote though.  - ATTRIBUTE  the following placeholders are recognized for   attribute values:  - CLASS for the class value in case a class attribute is set.  - ATTxyz with xyz a number from 1 to # of attributes in the  dataset, representing the value of indexed attribute.    Examples:  - extracting only mammals and birds from the 'zoo' UCI dataset:  (CLASS is 'mammal') or (CLASS is 'bird')  - extracting only animals with at least 2 legs from the 'zoo' UCI dataset:  (ATT14 >= 2)  - extracting only instances with non-missing 'wage-increase-second-year'  from the 'labor' UCI dataset:  not ismissing(ATT3)    Valid options are: |

---


|  |  |  |
| --- | --- | --- |
| **Overview** \| **Package** \| Class \| **Tree** \| **Deprecated** \| **Index** \| **Help** \| **Weka's home** | |  |
| **PREV PACKAGE**   **NEXT PACKAGE** | **FRAMES**    **NO FRAMES**     **All Classes** |  |


---
